# 2-[(1*H*-Benzimidazol-1-yl)meth­yl]phenol benzene hemisolvate

**DOI:** 10.1107/S1600536814000841

**Published:** 2014-01-22

**Authors:** Augusto Rivera, Leonardo Jiménez-Cruz, Michael Bolte

**Affiliations:** a Universidad Nacional de Colombia, Sede Bogotá, Facultad de Ciencias, Departamento de Química, Cra 30 No.45-03, Bogotá, Código Postal 111321, Colombia; b Institut für Anorganische Chemie, J.-W.-Goethe-Universität, Max-von-Laue-Strasse 7, Frankfurt/Main, D-60438, Germany

## Abstract

In the title solvate, C_14_H_12_N_2_O·0.5C_6_H_6_, the complete benzene molecule is generated by a crystallographic inversion centre. The dihedral angle between the planes of the benzimidazole moiety and the phenol substituent is 75.28 (3)°. In the crystal, O—H⋯N hydrogen bonds link the mol­ecules into parallel chains propagating along [100]. The mol­ecules are further connected by C—H⋯π inter­actions.

## Related literature   

For related structures, see: Cai *et al.* (2006 ▶); Rivera *et al.* (2012 ▶); Shi *et al.* (2011 ▶). For another synthesis procedure, see: Milata *et al.* (2001 ▶); Rivera *et al.* (2008 ▶). For the pharmacological use of benzimidazoles, see: Alamgir *et al.* (2007 ▶). For C—H⋯π inter­actions, see: Malathy Sony & Ponnuswamy (2005 ▶). 
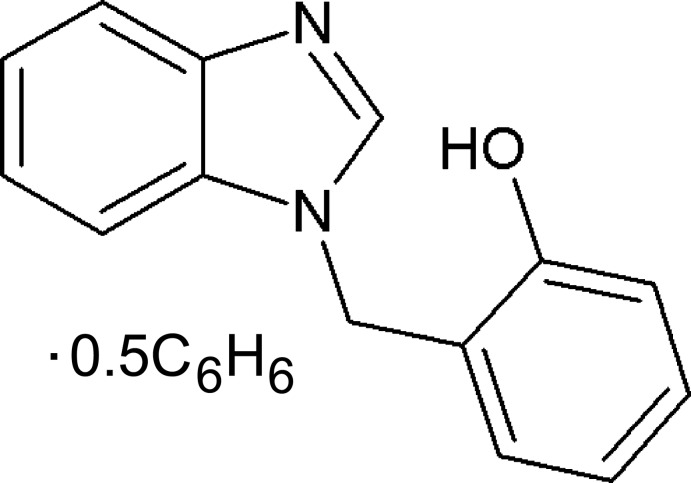


## Experimental   

### 

#### Crystal data  

C_14_H_12_N_2_O·0.5C_6_H_6_
*M* *_r_* = 263.31
Triclinic, 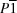

*a* = 8.9351 (11) Å
*b* = 9.3268 (10) Å
*c* = 9.9579 (11) Å
α = 73.098 (8)°
β = 69.124 (8)°
γ = 62.148 (8)°
*V* = 677.75 (15) Å^3^
*Z* = 2
Mo *K*α radiation
μ = 0.08 mm^−1^
*T* = 173 K
0.42 × 0.12 × 0.12 mm

#### Data collection  

Stoe IPDS II two-circle diffractometer
Absorption correction: multi-scan (*X-AREA*; Stoe & Cie, 2001 ▶) *T*_min_ = 0.967, *T*_max_ = 0.990
8981 measured reflections
2591 independent reflections
2314 reflections with *I* > 2σ(*I*)
*R*_int_ = 0.043

#### Refinement  

*R*[*F* ^2^ > 2σ(*F* ^2^)] = 0.040
*wR*(*F* ^2^) = 0.108
*S* = 1.12
2591 reflections
185 parameters
H atoms treated by a mixture of independent and constrained refinement
Δρ_max_ = 0.20 e Å^−3^
Δρ_min_ = −0.17 e Å^−3^

### 

Data collection: *X-AREA* (Stoe & Cie, 2001 ▶); cell refinement: *X-AREA*; data reduction: *X-AREA*; program(s) used to solve structure: *SHELXS97* (Sheldrick, 2008 ▶); program(s) used to refine structure: *SHELXL97* (Sheldrick, 2008 ▶); molecular graphics: *XP* in *SHELXTL-Plus* (Sheldrick, 2008 ▶); software used to prepare material for publication: *SHELXL97*.

## Supplementary Material

Crystal structure: contains datablock(s) I. DOI: 10.1107/S1600536814000841/sj5381sup1.cif

Structure factors: contains datablock(s) I. DOI: 10.1107/S1600536814000841/sj5381Isup2.hkl

Supporting information file. DOI: 10.1107/S1600536814000841/sj5381Isup3.cml

CCDC reference:

Additional supporting information:  crystallographic information; 3D view; checkCIF report

## Figures and Tables

**Table 1:** Hydrogen-bond geometry (Å, °) *Cg*1 is the centroid of the C2–C7 ring.

*D*—H⋯*A*	*D*—H	H⋯*A*	*D*⋯*A*	*D*—H⋯*A*
O1—H1⋯N2^i^	0.98 (2)	1.74 (2)	2.7200 (16)	174 (2)
C22—H22⋯*Cg*1	0.95	3.25	3.868	124

Symmetry code: (i) 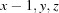.

**Fig. 1.**
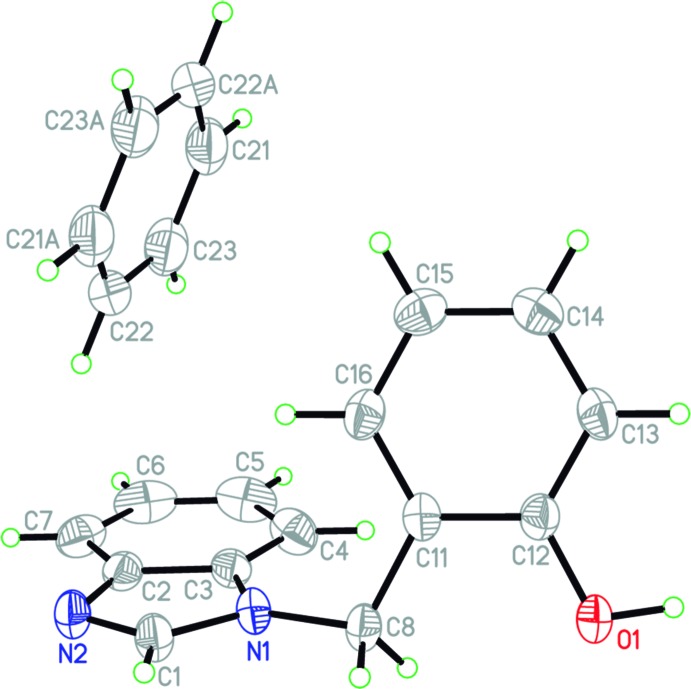
A view of the crystal structure of the title compound with the numbering scheme. Displacement ellipsoids are drawn at the 50% probability level.

**Fig. 2.**
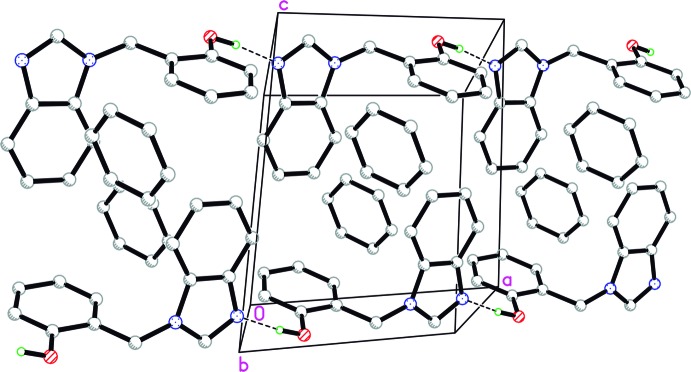
Packing of the molecules of the title compound viewed along the b axis. H atoms bonded to C atoms are omitted for clarity. O—H···N hydrogen bonds are drawn as dashed lines.

**Fig. 3.**
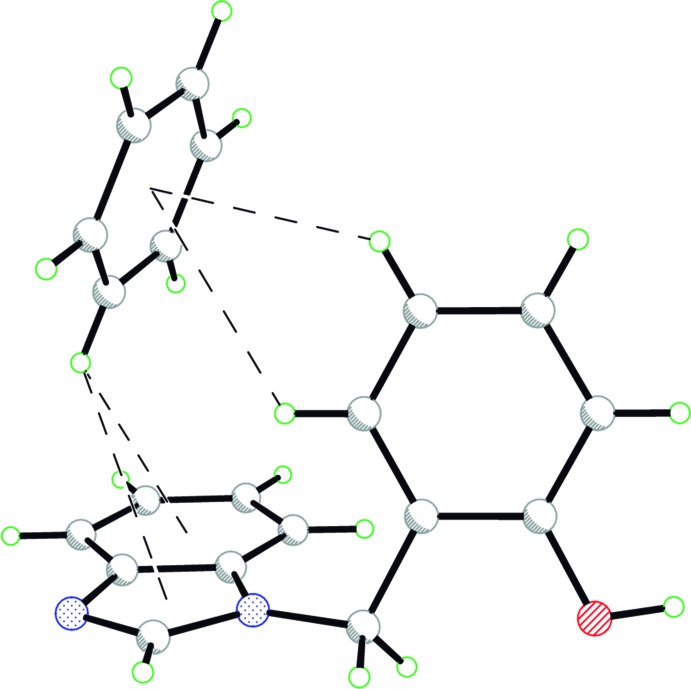
C—H···π interactions between 2-((1H-benzimidazol-1-yl)methyl)phenol and the benzene molecule.

## supplementary crystallographic information

### 1. Introduction

Appropriately substituted benzimidazole derivatives have found diverse therapeutic applications as anti­ulcer, anti­hypertensive, anti­viral, anti­fungal, anti­cancer, and anti­histaminic agents [Alamgir *et al.* 2007]. Although the synthesis of the title compound has been reported in the literature (Milata *et al.*, 2001; Rivera *et al.*, 2008), we have developed an alternative route to prepare this compound starting from *N*^1^,*N*^2^-bis­((1*H*-benzotriazol-1-yl)methyl)­benzene-1,2- di­amine.

The asymmetric unit contains one molecule of 2-((1H-benzimidazol-1-yl)methyl)­phenol and half a molecule of benzene (Fig. 1). The solvent molecule subtends a dihedral angle of 78.90 (6)° with respect to the phenol substituent and 70.99 (6)° with respect to the benzimidazole moiety, which suggest an edge-to-face (T-shaped) C—H···π inter­action according to the literature (Malathy Sony *et al.* 2005). The dihedral angle between the phenol substitutent and the benzimidazole ring [75.28 (3)°] is similar to the one in a related structure (Cai *et al.*, 2006). In the benzimidazole moiety, the bond distances and angles are in good agreement with those found in bis­(1*H*-benzimidazol-1-yl)methane monohydrate (Shi *et al.*, 2011), 1-(6-chloro­pyridin-3-yl­methyl)-1*H*-benzimidazole (Cai *et al.*, 2006) and (1*H*-benzimidazol-1-yl)methanol (Rivera *et al.*, 2012).

An inter­molecular hydrogen bond was observed in the crystal packing (Fig. 2) between the hydroxyl group of one molecule and a nitro­gen atom of another one (Table 1). The O—H distance is longer than in (1*H*-benzimidazol-1-yl)methanol [0.894 (19)Å] (Rivera *et al.*, 2012). However, the O···N distance [2.7200 (16)Å and 2.7355 (16)Å] and the O—H···N angle [174 (2)°, 173.8 (17)°] are similar in both structures. The O—H···N hydrogen bond connects the molecules forming chains running along the a-axis. The benzene molecule is linked to molecules of 2-((1H-benzimidazol-1-yl)methyl)­phenol via C—H···π inter­actions (Fig. 3) acting as both a donor and an acceptor, Table 1. The benzimidazole moiety also forms two C—H···π inter­actions to the phenol rings of neighbouring molecules. These values are is similar to the values reported for other C—H···π inter­actions (Malathy Sony *et al.* 2005).

### 2.1. Synthesis and crystallization

A mixture of phenol (0.282 g, 3.00 mmol) and *N*^1^,*N*^2^-bis­((1*H*-benzotriazol-1-yl)methyl)­benzene-1,2-di­amine (0.370 g, 1.00 mmol) was heated to 160 °C, after 5 minutes the mixture was cooled at room temperature until a sticky residue appeared. The product was purified by column chromatography using a mixture of benzene: ethyl acetate (80:20) as the mobile phase (yield 25 %, m.p.= 489-490 K). Single crystals were grown from a benzene:ethyl acetate solution by slow evaporation of the solvent at room temperature over a period of about one week.

### 2.2. Refinement

All H atoms were located in a difference map. The hydroxyl H atom was freely refined. H atoms bonded to C atoms were refined using a riding model, with secondary C—H = 0.99 Å and aromatic C—H = 0.95 Å and with *U*_iso_(H) = 1.2*U*_eq_(C).

### Crystal data

**Table:**

C_14_H_12_N_2_O·0.5C_6_H_6_	*Z* = 2
*M**_r_* = 263.31	*F*(000) = 278
Triclinic, *P*1	*D*_x_ = 1.290 Mg m^−^^3^
*a* = 8.9351 (11) Å	Mo *K*α radiation, λ = 0.71073 Å
*b* = 9.3268 (10) Å	Cell parameters from 14234 reflections
*c* = 9.9579 (11) Å	θ = 3.6–26.3°
α = 73.098 (8)°	µ = 0.08 mm^−^^1^
β = 69.124 (8)°	*T* = 173 K
γ = 62.148 (8)°	Needle, light brown
*V* = 677.75 (15) Å^3^	0.42 × 0.12 × 0.12 mm

### Data collection

**Table:**

Stoe IPDS II two-circle diffractometer	2314 reflections with *I* > 2σ(*I*)
Radiation source: Genix 3D IµS microfocus X-ray source	*R*_int_ = 0.043
ω scans	θ_max_ = 25.9°, θ_min_ = 3.9°
Absorption correction: multi-scan (*X-AREA*; Stoe & Cie, 2001)	*h* = −10→10
*T*_min_ = 0.967, *T*_max_ = 0.990	*k* = −11→11
8981 measured reflections	*l* = −12→12
2591 independent reflections	

### Refinement

**Table:**

Refinement on *F*^2^	0 restraints
Least-squares matrix: full	Hydrogen site location: mixed
*R*[*F*^2^ > 2σ(*F*^2^)] = 0.040	H atoms treated by a mixture of independent and constrained refinement
*wR*(*F*^2^) = 0.108	*w* = 1/[σ^2^(*F*_o_^2^) + (0.039*P*)^2^ + 0.238*P*] where *P* = (*F*_o_^2^ + 2*F*_c_^2^)/3
*S* = 1.12	(Δ/σ)_max_ < 0.001
2591 reflections	Δρ_max_ = 0.20 e Å^−^^3^
185 parameters	Δρ_min_ = −0.17 e Å^−^^3^

### Special details

**Table:**

Geometry. All e.s.d.'s (except the e.s.d. in the dihedral angle between two l.s. planes) are estimated using the full covariance matrix. The cell e.s.d.'s are taken into account individually in the estimation of e.s.d.'s in distances, angles and torsion angles; correlations between e.s.d.'s in cell parameters are only used when they are defined by crystal symmetry. An approximate (isotropic) treatment of cell e.s.d.'s is used for estimating e.s.d.'s involving l.s. planes.

### Fractional atomic coordinates and isotropic or equivalent isotropic displacement parameters (Å^2^)

**Table:**

	*x*	*y*	*z*	*U*_iso_*/*U*_eq_	
O1	0.26163 (13)	0.72436 (14)	0.00166 (12)	0.0358 (3)	
H1	0.155 (3)	0.706 (3)	0.023 (2)	0.064 (6)*	
N1	0.72313 (15)	0.66159 (14)	0.07264 (13)	0.0272 (3)	
N2	0.96889 (15)	0.66557 (15)	0.07838 (14)	0.0323 (3)	
C1	0.84911 (18)	0.71998 (18)	0.00788 (16)	0.0298 (3)	
H1A	0.8506	0.7939	−0.0809	0.036*	
C2	0.91796 (18)	0.56313 (17)	0.20031 (15)	0.0286 (3)	
C3	0.76391 (18)	0.55998 (16)	0.19817 (15)	0.0274 (3)	
C4	0.6810 (2)	0.46847 (19)	0.30695 (17)	0.0383 (4)	
H4	0.5766	0.4670	0.3040	0.046*	
C5	0.7585 (3)	0.3802 (2)	0.41906 (19)	0.0478 (4)	
H5	0.7057	0.3167	0.4963	0.057*	
C6	0.9130 (3)	0.3812 (2)	0.42267 (18)	0.0487 (5)	
H6	0.9627	0.3176	0.5018	0.058*	
C7	0.9953 (2)	0.47195 (19)	0.31466 (18)	0.0397 (4)	
H7	1.1000	0.4723	0.3180	0.048*	
C8	0.57443 (18)	0.69824 (19)	0.02012 (16)	0.0313 (3)	
H8A	0.5715	0.5940	0.0184	0.038*	
H8B	0.5908	0.7566	−0.0808	0.038*	
C11	0.40093 (17)	0.80188 (16)	0.11250 (15)	0.0270 (3)	
C12	0.24639 (18)	0.80968 (17)	0.09924 (15)	0.0274 (3)	
C13	0.08497 (18)	0.90253 (18)	0.18424 (16)	0.0322 (3)	
H13	−0.0200	0.9101	0.1733	0.039*	
C14	0.0764 (2)	0.98409 (18)	0.28484 (16)	0.0341 (3)	
H14	−0.0341	1.0448	0.3444	0.041*	
C15	0.2284 (2)	0.97727 (18)	0.29875 (16)	0.0344 (3)	
H15	0.2228	1.0335	0.3673	0.041*	
C16	0.38896 (19)	0.88763 (17)	0.21170 (16)	0.0309 (3)	
H16	0.4930	0.8848	0.2201	0.037*	
C21	0.4166 (2)	0.9279 (2)	0.62694 (19)	0.0456 (4)	
H21	0.3596	0.8781	0.7143	0.055*	
C22	0.6494 (2)	0.9049 (2)	0.4094 (2)	0.0455 (4)	
H22	0.7529	0.8387	0.3470	0.055*	
C23	0.5668 (2)	0.8322 (2)	0.5354 (2)	0.0474 (4)	
H23	0.6131	0.7161	0.5595	0.057*	

### Atomic displacement parameters (Å^2^)

**Table:**

	*U*^11^	*U*^22^	*U*^33^	*U*^12^	*U*^13^	*U*^23^
O1	0.0247 (5)	0.0495 (7)	0.0431 (6)	−0.0190 (5)	−0.0056 (4)	−0.0181 (5)
N1	0.0209 (6)	0.0305 (6)	0.0328 (6)	−0.0121 (5)	−0.0084 (5)	−0.0038 (5)
N2	0.0243 (6)	0.0380 (7)	0.0398 (7)	−0.0148 (5)	−0.0091 (5)	−0.0084 (5)
C1	0.0265 (7)	0.0340 (7)	0.0320 (7)	−0.0160 (6)	−0.0075 (6)	−0.0030 (6)
C2	0.0255 (7)	0.0274 (7)	0.0331 (7)	−0.0072 (5)	−0.0087 (6)	−0.0098 (6)
C3	0.0246 (7)	0.0246 (6)	0.0308 (7)	−0.0073 (5)	−0.0062 (5)	−0.0073 (5)
C4	0.0357 (8)	0.0307 (8)	0.0422 (9)	−0.0149 (6)	−0.0026 (7)	−0.0040 (6)
C5	0.0580 (11)	0.0309 (8)	0.0373 (9)	−0.0133 (8)	−0.0038 (8)	−0.0004 (7)
C6	0.0630 (12)	0.0327 (8)	0.0364 (9)	−0.0019 (8)	−0.0219 (8)	−0.0050 (7)
C7	0.0381 (8)	0.0359 (8)	0.0440 (9)	−0.0020 (7)	−0.0211 (7)	−0.0140 (7)
C8	0.0233 (7)	0.0378 (8)	0.0387 (8)	−0.0126 (6)	−0.0106 (6)	−0.0102 (6)
C11	0.0241 (7)	0.0272 (7)	0.0318 (7)	−0.0117 (5)	−0.0099 (5)	−0.0018 (5)
C12	0.0266 (7)	0.0298 (7)	0.0295 (7)	−0.0147 (6)	−0.0075 (5)	−0.0036 (5)
C13	0.0238 (7)	0.0357 (8)	0.0383 (8)	−0.0156 (6)	−0.0055 (6)	−0.0044 (6)
C14	0.0313 (8)	0.0318 (7)	0.0327 (8)	−0.0119 (6)	−0.0023 (6)	−0.0052 (6)
C15	0.0418 (8)	0.0294 (7)	0.0320 (7)	−0.0116 (6)	−0.0137 (6)	−0.0046 (6)
C16	0.0308 (7)	0.0294 (7)	0.0373 (8)	−0.0120 (6)	−0.0162 (6)	−0.0030 (6)
C21	0.0432 (9)	0.0642 (11)	0.0411 (9)	−0.0337 (9)	−0.0094 (7)	−0.0055 (8)
C22	0.0305 (8)	0.0586 (11)	0.0556 (10)	−0.0191 (8)	−0.0015 (7)	−0.0303 (9)
C23	0.0391 (9)	0.0384 (9)	0.0715 (12)	−0.0159 (7)	−0.0207 (8)	−0.0098 (8)

### Geometric parameters (Å, º)

**Table:**

O1—C12	1.3602 (17)	C8—H8A	0.9900
O1—H1	0.98 (2)	C8—H8B	0.9900
N1—C1	1.3531 (18)	C11—C16	1.390 (2)
N1—C3	1.3807 (19)	C11—C12	1.3999 (19)
N1—C8	1.4570 (17)	C12—C13	1.391 (2)
N2—C1	1.3101 (19)	C13—C14	1.386 (2)
N2—C2	1.390 (2)	C13—H13	0.9500
C1—H1A	0.9500	C14—C15	1.385 (2)
C2—C7	1.395 (2)	C14—H14	0.9500
C2—C3	1.398 (2)	C15—C16	1.387 (2)
C3—C4	1.390 (2)	C15—H15	0.9500
C4—C5	1.374 (3)	C16—H16	0.9500
C4—H4	0.9500	C21—C22^i^	1.370 (3)
C5—C6	1.398 (3)	C21—C23	1.383 (3)
C5—H5	0.9500	C21—H21	0.9500
C6—C7	1.379 (3)	C22—C21^i^	1.370 (3)
C6—H6	0.9500	C22—C23	1.375 (3)
C7—H7	0.9500	C22—H22	0.9500
C8—C11	1.5114 (19)	C23—H23	0.9500
			
C12—O1—H1	111.1 (12)	C11—C8—H8B	109.0
C1—N1—C3	106.30 (12)	H8A—C8—H8B	107.8
C1—N1—C8	126.83 (12)	C16—C11—C12	118.72 (13)
C3—N1—C8	126.86 (12)	C16—C11—C8	122.58 (12)
C1—N2—C2	104.48 (11)	C12—C11—C8	118.69 (12)
N2—C1—N1	114.07 (13)	O1—C12—C13	122.49 (12)
N2—C1—H1A	123.0	O1—C12—C11	117.60 (12)
N1—C1—H1A	123.0	C13—C12—C11	119.91 (13)
N2—C2—C7	130.19 (14)	C14—C13—C12	120.37 (13)
N2—C2—C3	109.62 (12)	C14—C13—H13	119.8
C7—C2—C3	120.19 (14)	C12—C13—H13	119.8
N1—C3—C4	131.74 (14)	C15—C14—C13	120.17 (13)
N1—C3—C2	105.52 (12)	C15—C14—H14	119.9
C4—C3—C2	122.74 (14)	C13—C14—H14	119.9
C5—C4—C3	116.22 (16)	C14—C15—C16	119.39 (13)
C5—C4—H4	121.9	C14—C15—H15	120.3
C3—C4—H4	121.9	C16—C15—H15	120.3
C4—C5—C6	121.83 (16)	C15—C16—C11	121.41 (13)
C4—C5—H5	119.1	C15—C16—H16	119.3
C6—C5—H5	119.1	C11—C16—H16	119.3
C7—C6—C5	121.91 (15)	C22^i^—C21—C23	119.45 (16)
C7—C6—H6	119.0	C22^i^—C21—H21	120.3
C5—C6—H6	119.0	C23—C21—H21	120.3
C6—C7—C2	117.10 (16)	C21^i^—C22—C23	120.50 (16)
C6—C7—H7	121.4	C21^i^—C22—H22	119.8
C2—C7—H7	121.4	C23—C22—H22	119.8
N1—C8—C11	112.97 (11)	C22—C23—C21	120.05 (17)
N1—C8—H8A	109.0	C22—C23—H23	120.0
C11—C8—H8A	109.0	C21—C23—H23	120.0
N1—C8—H8B	109.0		
			
C2—N2—C1—N1	−0.25 (16)	C3—C2—C7—C6	−0.1 (2)
C3—N1—C1—N2	0.43 (16)	C1—N1—C8—C11	−110.59 (16)
C8—N1—C1—N2	−179.40 (12)	C3—N1—C8—C11	69.62 (17)
C1—N2—C2—C7	−179.68 (15)	N1—C8—C11—C16	15.8 (2)
C1—N2—C2—C3	−0.02 (15)	N1—C8—C11—C12	−163.13 (12)
C1—N1—C3—C4	179.49 (15)	C16—C11—C12—O1	−179.84 (12)
C8—N1—C3—C4	−0.7 (2)	C8—C11—C12—O1	−0.84 (19)
C1—N1—C3—C2	−0.40 (14)	C16—C11—C12—C13	0.2 (2)
C8—N1—C3—C2	179.42 (12)	C8—C11—C12—C13	179.21 (13)
N2—C2—C3—N1	0.27 (15)	O1—C12—C13—C14	178.38 (13)
C7—C2—C3—N1	179.96 (12)	C11—C12—C13—C14	−1.7 (2)
N2—C2—C3—C4	−179.63 (12)	C12—C13—C14—C15	1.7 (2)
C7—C2—C3—C4	0.1 (2)	C13—C14—C15—C16	−0.3 (2)
N1—C3—C4—C5	−179.58 (14)	C14—C15—C16—C11	−1.2 (2)
C2—C3—C4—C5	0.3 (2)	C12—C11—C16—C15	1.2 (2)
C3—C4—C5—C6	−0.6 (2)	C8—C11—C16—C15	−177.73 (13)
C4—C5—C6—C7	0.6 (3)	C21^i^—C22—C23—C21	−0.3 (3)
C5—C6—C7—C2	−0.2 (2)	C22^i^—C21—C23—C22	0.3 (3)
N2—C2—C7—C6	179.51 (14)		

Symmetry code: (i) −*x*+1, −*y*+2, −*z*+1.

### Hydrogen-bond geometry (Å, º)

Cg1, Cg2, Cg3 and Cg4 are the centroids of the C2–C7, N1/N2/C1–C3, C21–C23/C21'–C23' and C11–C16 rings, respectively.

**Table:**

*D*—H···*A*	*D*—H	H···*A*	*D*···*A*	*D*—H···*A*
O1—H1···N2^ii^	0.98 (2)	1.74 (2)	2.7200 (16)	174 (2)
C22—H22···*Cg*1	0.95	3.25	3.868	124
C22—H22···*Cg*2	0.95	3.10	3.844	137
C15—H15···*Cg*3	0.95	3.06	3.761	132
C16—H16···*Cg*3	0.95	3.30	3.883	122
C1—H1*A*···*Cg*4^iii^	0.95	2.65	3.467	145
C5—H5···*Cg*4^iv^	0.95	3.17	3.922	138

Symmetry codes: (ii) *x*−1, *y*, *z*; (iii) −*x*+1, −*y*+2, −*z*; (iv) −*x*+1, −*y*+1, −*z*+1.
